# CYP450 Enzyme-Mediated Metabolism of TCAS and Its Inhibitory and Induced Effects on Metabolized Enzymes *in* *Vitro*

**DOI:** 10.3390/ijerph120910783

**Published:** 2015-09-02

**Authors:** Guolin Shen, Cheng Wang, Lili Zhou, Lei Li, Huiming Chen, Wenlian Yu, Haishan Li

**Affiliations:** Chinese Academy of Inspection and Quarantine, Beijing 100123, China; E-Mails: shenguolin801129@163.com (G.S.); wangz@aqsiqch.ac.cn (C.W.); zhoull@aqsiqch.ac.cn (L.Z.); lil@aqsiqch.ac.cn (L.L.); chenhm@aqsiqch.ac.cn (H.C.)

**Keywords:** thiacalix[4]arene tetrasulfonate, CYP450, hepatocyte, metabolism

## Abstract

In this study, we investigated the enzymes catalyzing the phaseⅠmetabolism of thiacalixarene (TCAS) based on *in vitro* system including cDNA-expressed P450 enzymes, human liver microsomes plus inhibitors and monoclonal antibodies. In addition, the inhibitory potential of TCAS on major CYP450 drug metabolizing enzymes (CYP1A2, CYP2C9, CYP2B6, CYP2D6 and CYP3A4) was assessed. The results showed that CYP1A2 and CYP2C9 mediated TCAS hydroxylation. IC_50_ values for TCAS in rat and human liver microsomes were greater than 50 µM, and it demonstrated a weak inhibition of rat and human CYP450 enzymes. Finally, sandwiched hepatocytes were used to evaluate the induction of CYP1A and CYP3A to define the function of TCAS *in vivo.* The results showed that incubation of TCAS at different concentrations for 72 h failed to induce CYP1A and CYP3A. However, incubation of the cells with 50 and 100 µM TCAS caused a profound decrease in the activities of CYP1A and CYP3A, which was probably due to cytotoxic effects, suggesting that exposure to TCAS might be a health concern.

## 1. Introduction

Thiacalixarene (TCAS) is a cyclic oligomer containing multiple phenol units bound by sulfur atoms bridging the *ortho* phenolic hydroxyl groups. It is considered the representative of the third-generation supramolecular receptor compounds [1]. Generally, thiacalixarene is categorized by the number of phenol units, for example, a thiacalixarene containing four phenol units is designated as thiacalixarene[4]. Its derivatives are generated by introducing different functional groups on the upper (phenyl ring groups) and the lower (phenolic hydroxyl groups) edges of its molecule. Synergistic non-covalent interactions lead to formation of ionic and molecular complexes of thiacalixarene and its derivatives [2]. Further, the lipid-soluble thiacalixarene and its derivatives may be rendered water-soluble by introducing specific acid radical groups, for e.g., hydrosoluble sulfonated thiacalixarene is derived by introducing four sodium sulfonate groups into the phenyl ring of TCAS. With the rapid industrialization and urbanization occurring in China, heavy metal pollution of the soil has reached a serious level, and is threatening the sustainable use of soil resources and the ecological safety of agricultural products. Currently, soil replacement, which is not effective, is the most used approach to remediate heavy metal-contaminated sites in China. Other methods are also used, but have limitations as well. For example, contaminants processed by chemical fixation still remain in the soil, which is an environmental hazard that needs monitoring and evaluation for long-term safety [1]. Bioremediation is extensively used because of its cost-effective features and environmental safety, although it is associated with low efficiency and long running cycles. In recent years, soil flushing technology has become an important chemical remediation method due to its advantages of stability and total effect, short cycle, user-friendly system, reduced waste, and extensive application [3].

In the early development of soil flushing technology, optimal decontamination rates were the focus. In recent years, to address the needs of environmental protection and sustainable development, the technology has gradually evolved in a green and environmentally-friendly direction. However, the current flushing agents are barely capable of simultaneously decontaminating heavy metal and organic pollutants in the soil. A few flushing agents for heavy metals are not selective, which may cause large collateral losses of plant nutrients. Therefore, exploration of flushing agents that specifically decontaminate metal ions is always the goal.

TCAS, the third-generation supramolecular receptor compound, has partial affinity for transition metals [4]. TCAS has very strong detection and binding affinity for metal ions of soft acids, such as Cd^2+^, Hg^+^, Pb^2+^, Sn^2+^, *etc*. However, its binding affinity is extremely weak for metal ions of bases, such as K^+^, Na^+^, Ca^2+^ and Mg^2+^. Previous studies indicated that TCAS not only has a flushing effect similar to EDTA for heavy metals such as Cd and Cu, but also avoids the massive loss of K^+^, Na^+^, Ca^2+^ and Mg^2+^, justifying its use in soil remediation. Although no TCAS biosafety report is available, reports are available for calixarenes containing similar molecular structure and derivatives. In 2000, Perret first reported that phosphated calixarenes had no toxic side effects on the growth of human fibrinogen cells [4]. Da Silva *et al.* reported that the solubility of red blood cells was not affected with sulfonated calixarene and its three derivatives with single substitution by phenolic hydroxyl groups at a concentration of 200 mmol·L^−1^ [5]. Sulfonated calixarene did not trigger an immune response and was nontoxic below 50 mmol·L^−1^ [6]. Anthony *et al*. studied the acute toxicity of sulfonated calixarene in mice using a ^35^S isotope tracer method. The results suggested that sulfonated calixarene neither passed the blood-brain barrier nor entered the muscle cells in mice, but was rapidly excreted via urine. No sulfonated calixarene accumulated in the liver or spleen. Sulfonated calixarene at levels below 100 mg·kg^−1^ was non-toxic for mice [7]. In the absence of any biosafety assessment of TCAS or evaluation of the potential ecological risks of TCAS residues in soil after leaching, the safety risks of TCAS residues in environment were explored in this study. We elucidated its metabolic mechanisms using liver microsomes and recombinant enzymes and evaluated its effects on CYP450 enzyme in primary liver cells.

## 2. Materials and Methods

### 2.1. Chemicals

Thiacalix[4]arene tetrasulfonate (TCAS) with purity greater than 98% was purchased from TCI America (city, state abbrev if USA, country). α-Naphthoflavone and β-NADPH were purchased from Roche Molecular Biochemicals (Basel, Switzerland). Recombinant human cytochromes P450 (CYP1A2, 3A5, 2C9, 2D6, 2E1 and 3A4), pooled human liver microsomes (HLMs) and CYP450 monoclonal antibodies (CYP1A2, 2C9, 2D6 and 3A4) were provided by BD Gentest (Woburn, MA, USA). All other reagents and solvents were of analytical grade and commercially available.

### 2.2. Quantification of TCAS

The quantitative determination of TCAS was conducted using a new method. A Thermo TSQ Vantage triple quadrupole mass spectrometer (Thermo Fisher Scientific, San Jose, CA, USA) equipped with a Waters ACQUITY Ultra Performance HPLC system and a Thermo Syncronis C18 column (100 mm × 2.1 mm, 1.9 µm I.D.) was used in this study. The mobile phase consisted of 0.1% formic acid water solution (A) and acetonitrile containing 0.1% formic acid (B). The gradient program used was as follows: 0 min 10% B, 0–1 min 20% B, 1–1.5 min 85% B, 1.5–2 min 85% B, and 2–3 min 10% B, for a total duration of 4.5 min. The column temperature was set at 25 °C and the flow rate was 0.25 mL·min^−1^. For quantification, the instrument was operated in the ESI positive ion multiple reaction monitoring (SIM) mode with the following optimized MS/MS conditions: transfer capillary temperature of 350 °C; spray voltage of 3500 V. The selected transitions were 271.6 for TCAS and 260 for PRO (IS). The linear quantitation ranges for TCAS ranged from 50 to 500 μg/mL. The lower limit of detection of TCAS was 50 μg/mL.

### 2.3. TCAS Incubation with cDNA-Expressed P450 Enzymes

A standard incubation mixture (200 µL) consisted of 2 pmol/mL of complementary DNA (cDNA)-expressed human cytochrome P450 enzymes, 100 µM of TCAS, 3.3 mM of MgCl_2_ and 100 mM of sodium phosphate buffer (pH 7.4). This experiment was performed in triplicate. After 5 min of preincubation at 37 °C, the reaction was initiated by adding 10 μL of 20 mM NADPH (final concentration 1.0 mM) and continued for 60 min with gentle shaking. Incubations in the absence of NADPH served as controls. Reactions were terminated using 400 μL of ice-cold stop solution (a 1:1 (v:v) mixture of acetonitrile and methanol) containing 50 ng/mL tolbutamide (IS), vortexing for 30 sec, and centrifugation at 16,000 g for 10 min. The supernatant was collected and analyzed by an LC-MS/MS method described above.

### 2.4. Incubation with Human Liver Microsomes Plus Inhibitors

All human liver microsomal (0.4 mg/mL) incubations were conducted in 100 mM phosphate buffer, pH 7.4, initiated by the addition of NADPH (1 mM final concentration) for 5 min before incubation with TCAS (100 µM) and inhibitor of specific isoform of CYP450. The following CYP450 isoform-selective inhibitors were used: α-naphthoflavone (6 µM, CYP1A2); sulfaphenazole (10 µM, CYP2C9); tranylcypromine (600 µM, CYP2C19); quinidine (6 µM, CYP2D6) and ketoconazole (6 µM, CYP3A4) [8,9,10]. Inhibitor stock solutions prepared in appropriate solvents accounted for 0.4% organic solvent in the final incubation for 30 min at 37 °C with gentle shaking before stopping with 400 μL ice-cold stop solution (1:1 (v:v) mixture of acetonitrile and methanol) containing 50 ng/mL tolbutamide (IS). After vortexing for 30 seconds, and centrifugation at 16,000 g for 10 min, the supernatant was collected and analyzed by LC-MS/MS as described above. Control incubations with inactive HLMs or without inhibitor were conducted. All incubations were conducted in triplicate.

### 2.5. Incubation with Human Liver Microsomes Plus Monoclonal Antibodies

The antibodies were preincubated with microsomes in an ice-bath for 20min, which contained enzymes of CYP1A2, CYP2C9, CYP2D6 and CYP3A4. Then, they were incubated with TCAS (100 µM) and the NADPH generating system at 37 °C for 30 min before stopping with 400 μL ice-cold stop solution (1:1 (v:v) mixture of acetonitrile and methanol) containing 50 ng/mL tolbutamide (IS). After vortexing for 30 seconds, and centrifugation at 16,000 g for 10 min, the supernatant was collected and analyzed by LC-MS/MS. Control incubations without monoclonal antibodies were conducted. All incubations were conducted in triplicate.

### 2.6. TCAS Inhibition of CYP450

The inhibitory effects of TCAS on the activities of CYP1A2, CYP2C9, CYP2D6, CYP2B6 and CYP3A were tested in RLMs and HLMs using probes selective for each isoform. The reaction probes used were as follows: phenacetin for CYP1A2; tolbutamide for CYP2C9; dextromethorphan for CYP2D6; midazolam for CYP3A and bupropion for CYP2B6. Incubation conditions specific to each isoform were linear with time, substrate, and protein concentrations as detailed in our previous studies. Isoform-specific substrate probes were incubated in duplicate at 37 °C with RLMs and HLMs and an NADPH-generating system in the absence (control) or presence of various concentrations (0.5, 1.5, 5, 15, 50, 100, and 500 µM) of TCAS. A 5-min pre-incubation was carried out before the reaction was initiated by adding 0.4 mg/mL of RLMs and HLMs. After incubation for 30 min, the reaction was terminated with 400 μL of ice-cold stop solution. After vortexing for 30 seconds, and centrifugation at 16,000 g for 10 minutes, the supernatant was collected and analyzed by LC-MS/MS [11].

### 2.7. Hepatocyte Culture

Rat primary hepatocytes were prepared according to a modified, two-step collagenase perfusion method [12]. Viability of hepatocyte was determined by Trypan Blue exclusion. Only those hepatocyte preparations with viability greater than 90% were used for further studies. Hepatocyte suspensions were prepared with DMEM-F12 containing 10% FBS, 100 U/mL penicillin, 100 µg/mL streptomycin, 1 µM DEX and 4 µg/mL insulin. Then, hepatocyte suspensions were seeded into the pre-coated wells at a density of 1 × 10^6^ cells/well in 24-well plates. Cells were incubated at 37 °C in a humidified incubator with 95% O_2_/5% CO_2_ and allowed to attach for 4 h, during which the medium was aspirated to remove unattached cells and fresh medium was added. Twenty-four hours later, cells were overlaid with BD Matrigel basement membrane matrix at a concentration of 0.25 mg/mL in 0.5 mL of ice-cold DMEM for an additional 24 h at 37 °C in a humidified incubator with 95% O_2_/5% CO_2_. Subsequently, cells were treated with TCAS (1, 5, 10, 50, 100 µM), phenobarbital (PB, 1 mM) and 3- methylcholanthrene (3-MC, 2.5 µM) from 24 to 96 h in culture. Medium was changed daily. On day 4, the cultured hepatocytes were washed twice with Hank’s balanced salt solution containing 25 mM HEPES and incubated with 0.5 mL of the same buffer containing 50 μM phenacetin and 5 μM midazolam at 37 °C for 1h. At the end of incubation, 0.5 mL aliquots of the media were transferred to 1.5-mL test tubes and stored at −20 °C until analysis. All incubations were conducted in triplicate.

## 3. Data Analysis

Results were analyzed by a two-factor ANOVA. A *p* < 0.05 was considered statistically significant. The inverse correlation between the formation of acetaminophen and 1-hydroxymidazolam in untreated hepatocytes and the fold increase in induced hepatocytes, was determined with regression analysis using SPSS. Microsoft Excel (Microsoft, Redmond, WA, USA) was used to calculate IC_50_ estimates by linear transformation of the raw data. The data were corrected for both background and control activities.

## 4. Results

### 4.1. Metabolic Enzyme Phenotyping of TCAS

**Figure 1:**
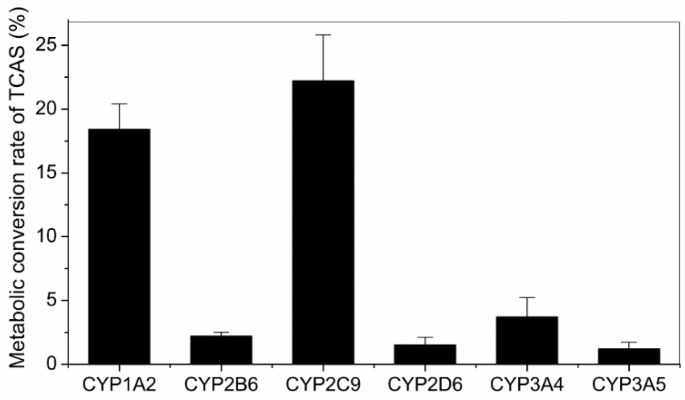
Metabolic conversion rate of TCAS in cDNA-expressed P450 enzymes. Values are presented as M ± SD (n = 3).

A panel of recombinant human metabolic enzymes (CYPs) was screened for activity against 100 μM TCAS. To account for drift in signal and spontaneous parent loss, each time point was compared with a TCAS sample (without enzyme) incubated under identical conditions. After 1-hour incubation, only CYP1A2 and 2C9 showed significant activity as measured by the conversion of TCAS (18.4% and 14.7%) (Figure 1). In contrast, other enzymes had minimal effect on TCAS metabolism. Therefore, the two enzymes contributed the primary metabolism of TCAS.

### 4.2. Inhibition with Selective Chemical Inhibitors and Monoclonal Antibodies

**Figure 2:**
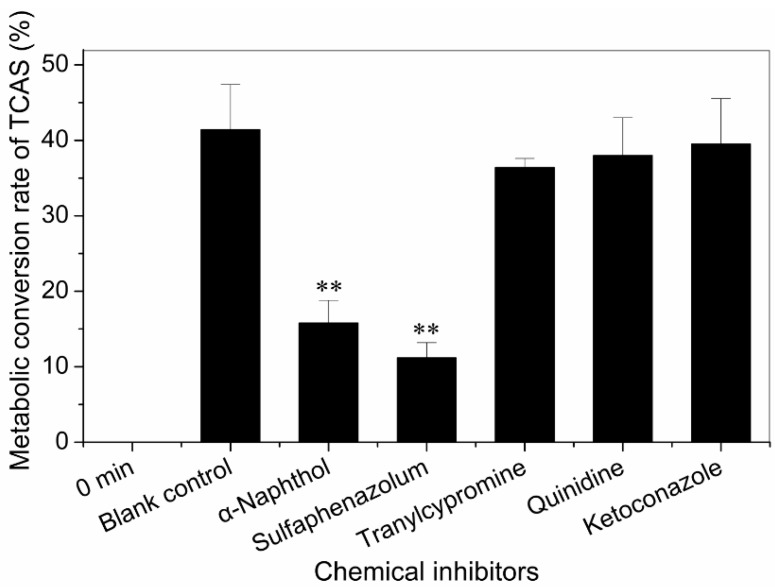
Metabolic conversion rate of TCAS in human liver microsomes plus inhibitors. Values are presented as M ± SD (n = 3). (* *p* < 0.05, ** *p* < 0.01, compared with blank control).

**Figure 3:**
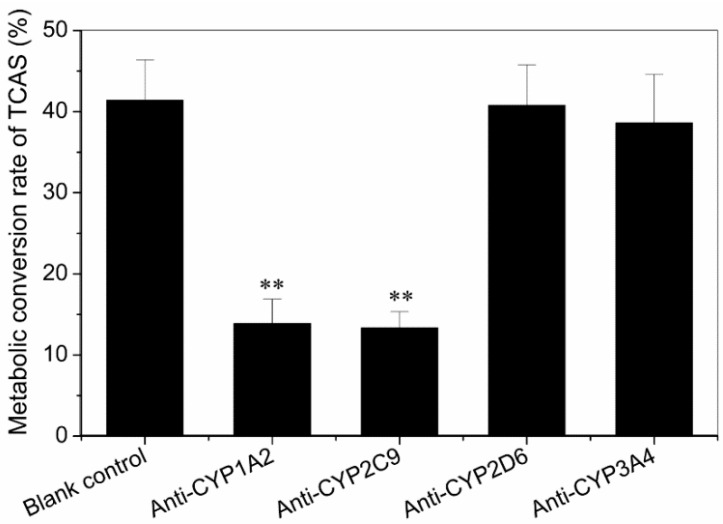
Metabolic conversion rate of TCAS in human liver microsomes plus monoclonal antibodies. Values are presented as M ± SD (n = 3). (* *p* < 0.05, ** *p* < 0.01, compared with blank control).

α-Naphthoflavone (6 µM) and sulfaphenazole (60 µM) inhibited TCAS formation by a mean 54.9% (*p* < 0.01) and 51.7% (*p* < 0.01), respectively, at a substrate concentration of 100 µM (Figure 2). Of the four antibodies tested, only anti-CYP1A2 and 2C9 substantially inhibited the formation of TCAS in HLMs (Figure 3). In addition, no appreciable effect was seen for anti-CYP2D6 and 3A4. The results showed that CYP1A2 and 2C9 were the enzymes which metabolized the TCAS.

### 4.3. IC_50_ of TCAS Inhibition

The potency of TCAS inhibition was determined on the basis of concentration-inhibition curves of the five CYP isoforms. As shown in Table 1, TCAS was not a strongly inhibitor of CYP1A2, CYP2D6, CYP3A4, CYP2B6 and CYP2C9. The IC_50_ values in RLMs or HLMs were all greater than 50 µM, suggesting that TCAS weakly inhibited rat and human CYP450 enzymes.

**Table 1:** Inhibitory potencies of TCAS on the activities of CYP450 enzymes in rat and human liver microsomes.

IC_50_(μM)	1A2	2C9	2B6	2D6	3A4
Rat	535.9 ± 10.3	382.8 ± 23.5	1696.0 ± 102.6	195.8 ± 17.6	2416.0 ± 234.6
Human	764.2 ± 50.4	777.6 ± 46.9	487.0 ± 52.1	278.4 ± 24.6	446.9 ± 48.2

Values are presented as M ± SD (n = 3).

### 4.4. Effect of TCAS on CYP1A and CYP3A Enzymatic Activity

**Figure 4:**
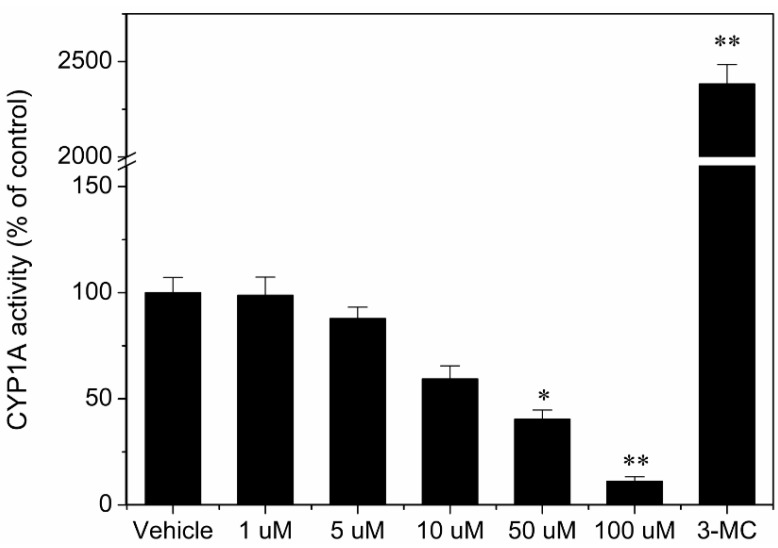
Induction of CYP1A activity in rat hepatocytes by TCAS.

**Figure 5:**
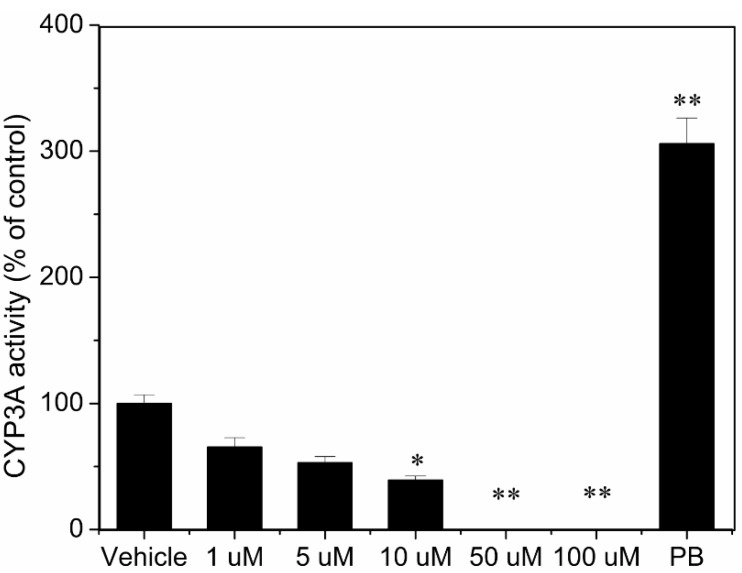
Induction of CYP3A activity in rat hepatocytes by TCAS. Values are presented as M ± SD (n = 3 rats). * *p* < 0.05 and ** *p* < 0.01 indicate statistically significant differences when compared to vehicle-treated cell.

The degree of CYP1A and CYP3A induction in rat hepatocytes was determined by measuring CYP1A and CYP3A enzymatic activity. Twenty-four hours after seeding, hepatocytes from three different rats were incubated for an additional 72 h in the presence of vehicle, 1, 5, 10, 50, 100 µM TCAS, 1 mM PB and 2.5 µM 3-MC, respectively. The CYP1A and CYP3A enzymatic activity in these cells was compared with the activity in control cells (0.1% DMSO) or cells incubated in the presence of a typical inducer of CYP1A (3-MC) or CYP3A (PB). Cells incubated in the presence of 2.5 µM 3-MC and 1 mM PB demonstrated a mean 23.8-fold and 5.1-fold induction of CYP1A and CYP3A activity, respectively, compared with control cells. In contrast, TCAS failed to induce CYP1A and CYP3A at different concentrations for 72 h (Figure 4 and Figure 5). Notably, incubation of the cells with 50 and 100 µM TCAS caused a profound decrease in the CYP1A and CYP3A activity, probably due to cytotoxic effects (data not shown). The four sulfonic acid groups in TCAS strongly denatured cellular proteins.

## 5. Discussion

Incubation of liver microsomes is generally used to study drug metabolism *in vitro*, as recommended by the relevant guidelines of the United States [13]. The liver microsomes consist of a variety of enzymes. We selected tolbutamide, dextromethorphan, phenacetin, midazolam and bupropion, which were extensively used as probe substrates for CYP2C9, CYP2D6, CYP1A2, CYP3A4 and CYP2B6, respectively, in this study [14,15,16]. Our study suggested that TCAS was metabolized via CYP1A2 and CYP2C9, with a weak inhibition against rat CYP1A2, CYP2C9 and CYP2D6 activity, but not against activities of rat CYP2B and CYP3A. It also exhibited weak inhibition against the activities of human CYP1A2, CYP2C9, CYP2B6, CYP2D6 and CYP3A4 based on the IC_50_ values (Table 1). The results suggested that TCAS was not a strong inhibitor of any of the CYP enzymes across different species. Therefore, TCAS appears to be relatively safe in terms of CYP-mediated drug interactions.

CYP450 induction has been investigated in rat hepatocytes [17,18]. This study presented the first report of induction of rat CYP450s by TCAS. The experiments with hepatocytes from different animal species were performed under near-identical conditions, with minimal differences attributable to experimental conditions. The results of the current study revealed that TCAS had no effect on activities of CYP1A and CYP3A when incubated in sandwiched cultured rat hepatocytes for three days. Notably, TCAS (>50 µM) had inhibitory effect on activities of CYP1A and CYP3A, suggesting that TCAS strongly affected the cell physiology. Therefore, human exposure to TCAS is associated with health risks.

## 6. Conclusions

In conclusion, TCAS was metabolized through CYP1A2 and CYP2C9, which was confirmed by a series of *in vitro* studies. In addition, TCAS inhibited activities of CYP1A, CYP2C and CYP2D, but not CYP2B and CYP3A in rat liver microsomes. It also exhibited weak inhibition against the activities of CYP1A2, CYP2C9, CYP2B6, CYP2D6 and CYP3A4 in human liver microsomes *in vitro*. Taken together, the results suggested that TCAS manifested weak interactions with drugs that are metabolized by different CYP450 enzymes, as discussed above. Our results revealed that TCAS had no effect on activities of CYP1A and CYP3A. Conversely, it exerted inhibitory effect on activities of CYP1A and CYP3A when the concentration of TCAS was greater than 50 µM, suggesting that human exposure to TCAS is not entirely safe.
